# A new species and first record of the genus *Procerobaetis* Kaltenbach & Gattolliat, 2020 (Ephemeroptera, Baetidae) from Thailand

**DOI:** 10.3897/zookeys.1023.61081

**Published:** 2021-03-10

**Authors:** Chanaporn Suttinun, Thomas Kaltenbach, Jean-Luc Gattolliat, Boonsatien Boonsoong

**Affiliations:** 1 Animal Systematics and Ecology Speciality Research Unit (ASESRU), Department of Zoology, Faculty of Science, Kasetsart University, Bangkok 10900, Thailand Kasetsart University Bangkok Thailand; 2 Museum of Zoology, Palais de Rumine, Place Riponne 6, CH-1005 Lausanne, Switzerland Museum of Zoology Lausanne Switzerland; 3 University of Lausanne (UNIL), Department of Ecology and Evolution, CH-1015 Lausanne, Switzerland University of Lausanne Lausanne Switzerland

**Keywords:** Mayfly, taxonomy, Southeast Asia

## Abstract

The genus *Procerobaetis* Kaltenbach & Gattolliat, 2020 is reported for the first time from Thailand, and *Procerobaetis totuspinosus***sp. nov.** is described as a new species based on larvae. It can be easily distinguished from other known *Procerobaetis* species by the presence of triangular spines at the posterior margin of tergites VI–IX. COI sequences were obtained from all known species. The genetic distances (Kimura 2-parameter) between the new species and the other species are between 20% and 23%. The morphological characters of the new species and its closely related species are discussed; larval key to all species of the genus *Procerobaetis* is also provided.

## Introduction

The family Baetidae is the most diverse mayﬂy family at the species level, accounting for about 30% of all mayfly species worldwide (Barber-James et al. 2008; Jacobus et al. 2019). Approximately nine genera and 13 species have been recorded from Thailand (Müller-Liebenau and Heard 1979; Thomas 1992; Sites et al. 2001; Boonsoong et al. 2004; Kluge 2016; Kluge and Novikova 2017; Sutthinun et al. 2018; Kluge et al. 2020; Kluge and Suttinun 2020). In the past decade, knowledge about the diversity of Baetidae in Thailand has continued to increase, and more taxa have been described, including *Procloeon* Bengtsson, 1915 (Tungpairojwong and Bae 2015; Kluge 2016), *Anafroptilum* Kluge, 2012 (Kluge and Novikova 2017), *Platybaetis* Müller-Liebenau, 1980 (Sutthinun et al. 2018), *Centroptella* Braasch & Soldán, 1980 (Kluge et al. 2020), *Indocloeon* Müller-Liebenau, 1982 (Kluge and Suttinun 2020), as well as the genus *Cymbalcloeon* Suttinun, Gattolliat & Boonsoong, 2020, which is endemic to this country (Suttinun et al. 2020).

Recently, the genus *Procerobaetis* Kaltenbach & Gattolliat, 2020 of Baetidae was discovered from Southeast Asia. It presently contains three species distributed in Indonesia: Sumatra (*P. leptobranchius* and *P. petersorum*) and the Philippines (*P. freitagi*), as described by Kaltenbach et al. (2020). Here, we describe a new species of *Procerobaetis* from Thailand based on material collected from the northern part of the country. This is one of the results of the first mass survey of the family Baetidae in Thailand. Materials were collected from May 2017 to June 2020 in 105 localities from all parts of Thailand, mainly from the southern and the western parts. As the family Baetidae is still poorly known in Thailand, our study will help to improve this situation. The latest new genus of this family was also described based on the same collection campaign (Suttinun et al. 2020).

## Materials and methods

The specimens were collected from streams and wadeable rivers from the northern part of Thailand. They were subsequentely preserved in 95% ethanol. Larval dissection was performed in Cellosolve, with subsequent mounting on slides with Euparal. Measurements (given in mm) and photographs were taken using a Visionary LK System (Dun, Inc., USA). All drawings were made with the aid of a camera lucida attached to a compound microscope. For scanning electron microscopy (SEM), specimens (head, legs, tergites, paraproct, caudal firmaments) were dried in a critical point drier (CPD7501) and coated with gold (Sputter Coater SC7620). The SEM photographs were observed with a FEI Quanta 450 SEM. Final plates were prepared with Adobe Photoshop CC 2020.

The DNA was extracted using non-destructive methods, allowing subsequent morphological analysis (see Vuataz et al. 2011 for details). The COI (658 bp fragment of the mitochondrial gene cytochrome oxidase subunit 1) were amplified using the primers LCO1490 and HCO2198 (Folmer et al. 1994). The polymerase chain reaction (PCR) conditions and procedure were performed as described by Kaltenbach et al. (2020). Sequencing was done with Sanger’s method (Sanger et al. 1977). The genetic distances between species was performed using Kimura-2-parameter distances (K2P, Kimura 1980), calculated with the program MEGA X (Kumar et al. 2018). The GenBank accession numbers are given in Table 1.

**Table 1.** Sequenced specimens of the genus *Procerobaetis* (in bold new sequence).

Species	Locality	GenBank Accession Number (GenSeq Nomenclature)
***P. totuspinosus* sp. nov.**	**Chiang Mai, Thailand**	**MW549043 (genseq-2 COI)**
*P. leptobranchius*	Sumatra, Indonesia	MN453816, MN453817
*P. petersorum*	Sumatra, Indonesia	MN453818, MN453814
*Procerobaetis* sp. C	Sumatra, Indonesia	MN453815
*P. freitagi*	Mindoro, Philippines	MN453819

The distribution map was generated with the software SimpleMappr (Shorthouse 2010).

The material is deposited in the collection of the Zoological Museum at Kasetsart University in Bangkok, Thailand (**ZMKU**) and at the Museum of Zoology in Lausanne, Switzerland (**MZL**).

## Taxonomy

**Order Ephemeroptera Hyatt & Arms, 1891**

**Family Baetidae Leach, 1815**

### Genus *Procerobaetis* Kaltenbach & Gattolliat, 2020

#### Procerobaetis totuspinosus

Taxon classificationAnimaliaEphemeropteraBaetidae

Suttinun, Kaltenbach & Boonsoong sp. nov.

6F586DA9-FBD2-562C-BF95-5C7A4229CEFF

http://zoobank.org/964C6E8F-8417-4F94-ABE1-40D28F07CD4D

**Figure 1.**
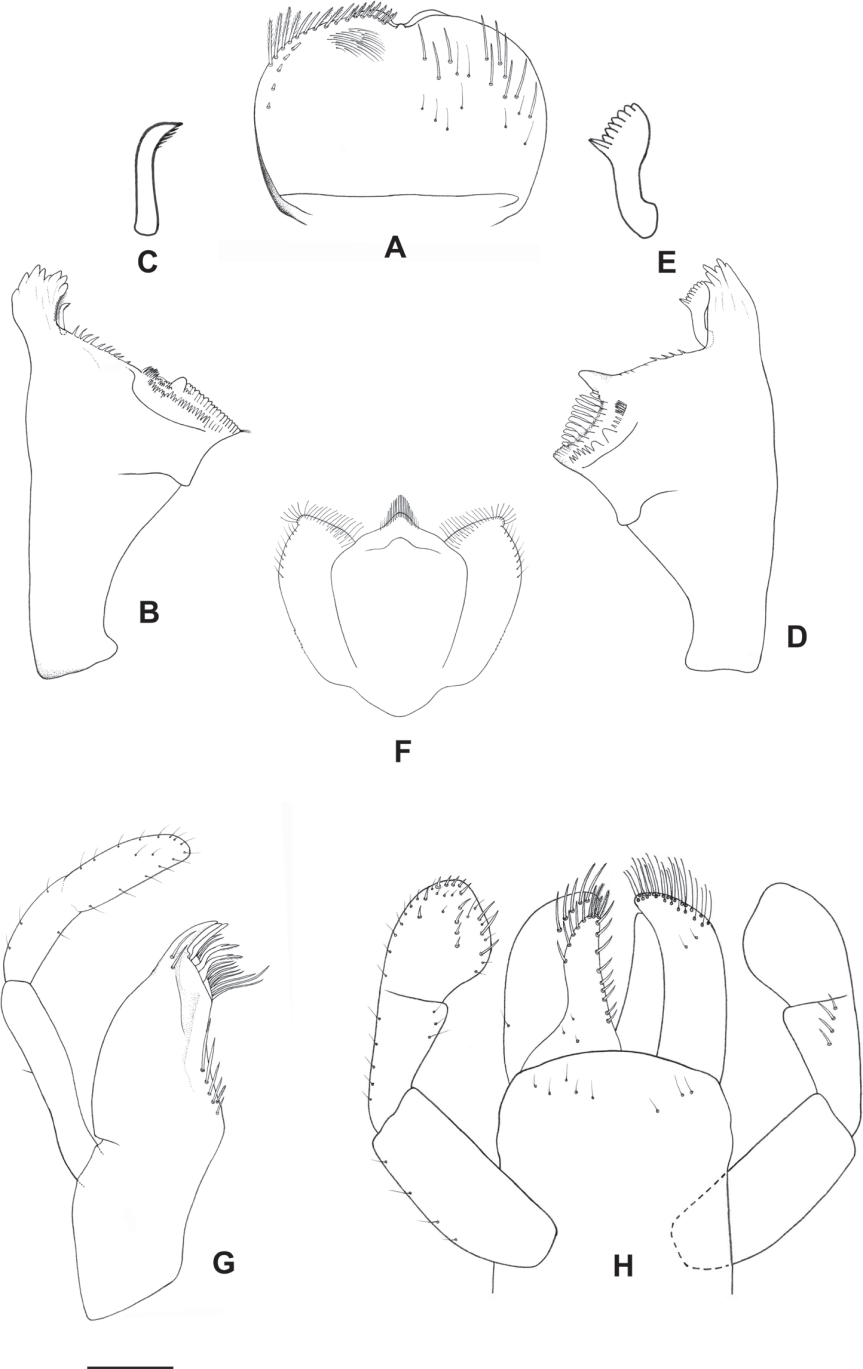
*Procerobaetis totuspinosus* sp. nov., larval morphology **A** labrum **B** right mandible **C** right prostheca **D** left mandible **E** left prostheca **F** hypopharynx **G** maxilla **H** labium. Scale bar: 0.1 mm.

**Figure 2.**
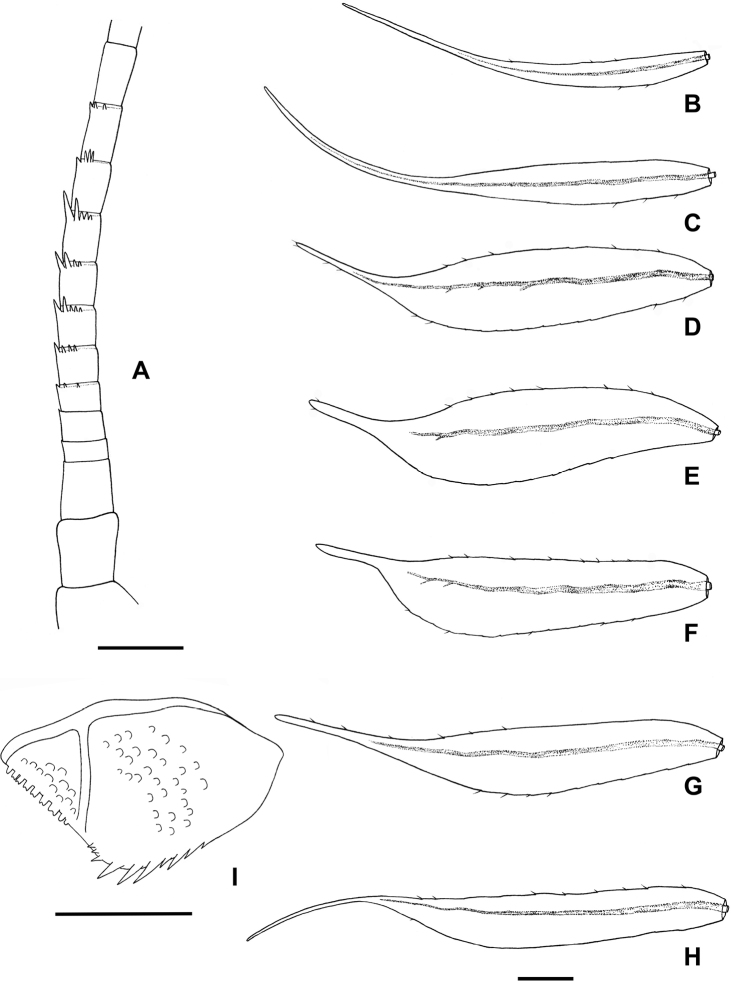
*Procerobaetis totuspinosus* sp. nov., larval morphology **A** antenna **B** gill I **C** gill II **D** gill III **E** gill IV **F** gill V **G** gill VI **H** gill VII **I** paraproct. Scale bars: 0.1 mm.

**Figure 3.**
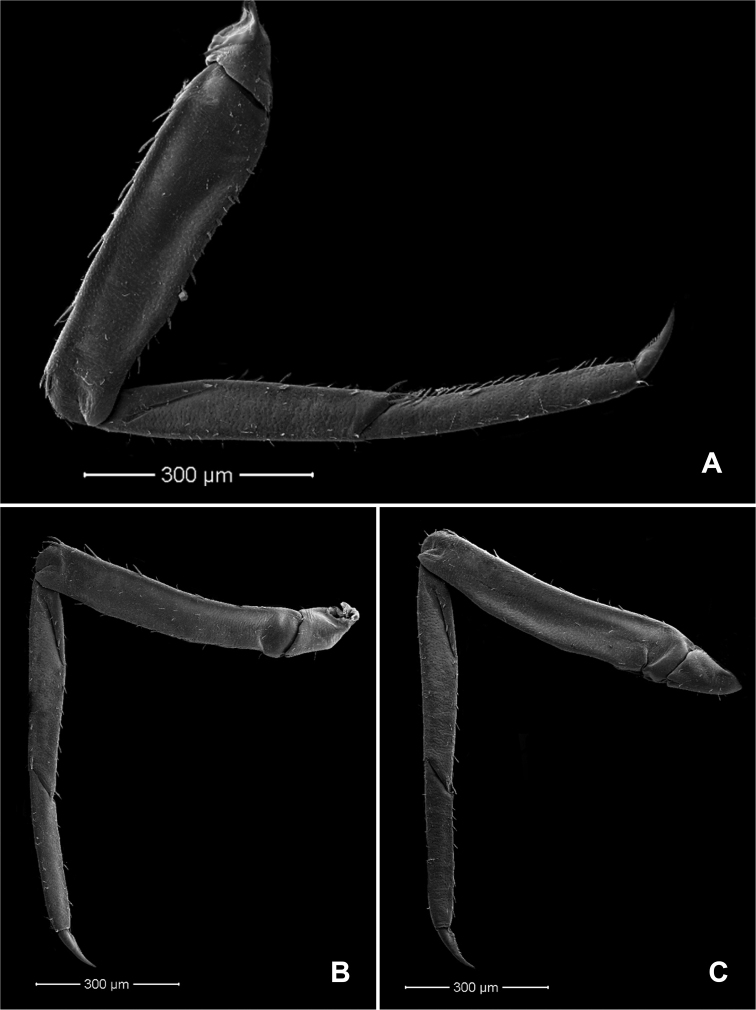
*Procerobaetis totuspinosus* sp. nov., SEMs of legs **A** foreleg **B** middle leg **C** hind leg.

**Figure 4.**
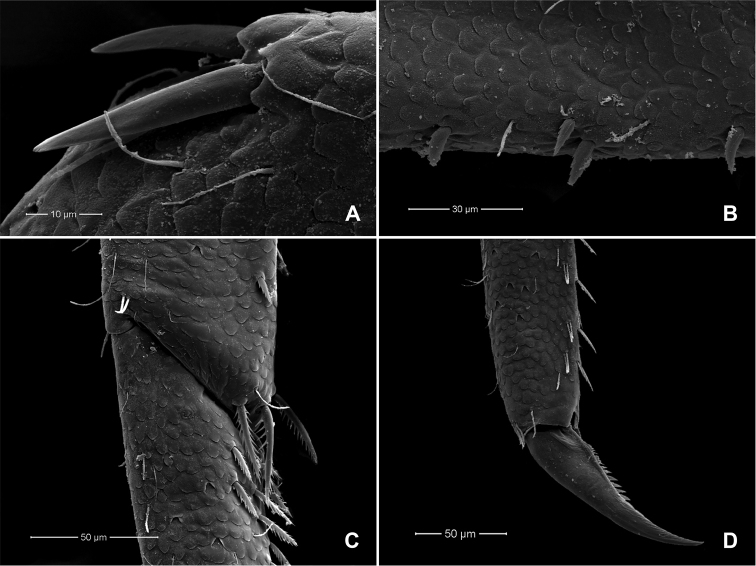
*Procerobaetis totuspinosus* sp. nov., SEMs of foreleg **A** setae at dorsal margin of femur apex **B** setae near ventral margin of femur **C** setae at ventral margin of tibia apex and proximal tarsus **D** fore claw.

**Figure 5.**
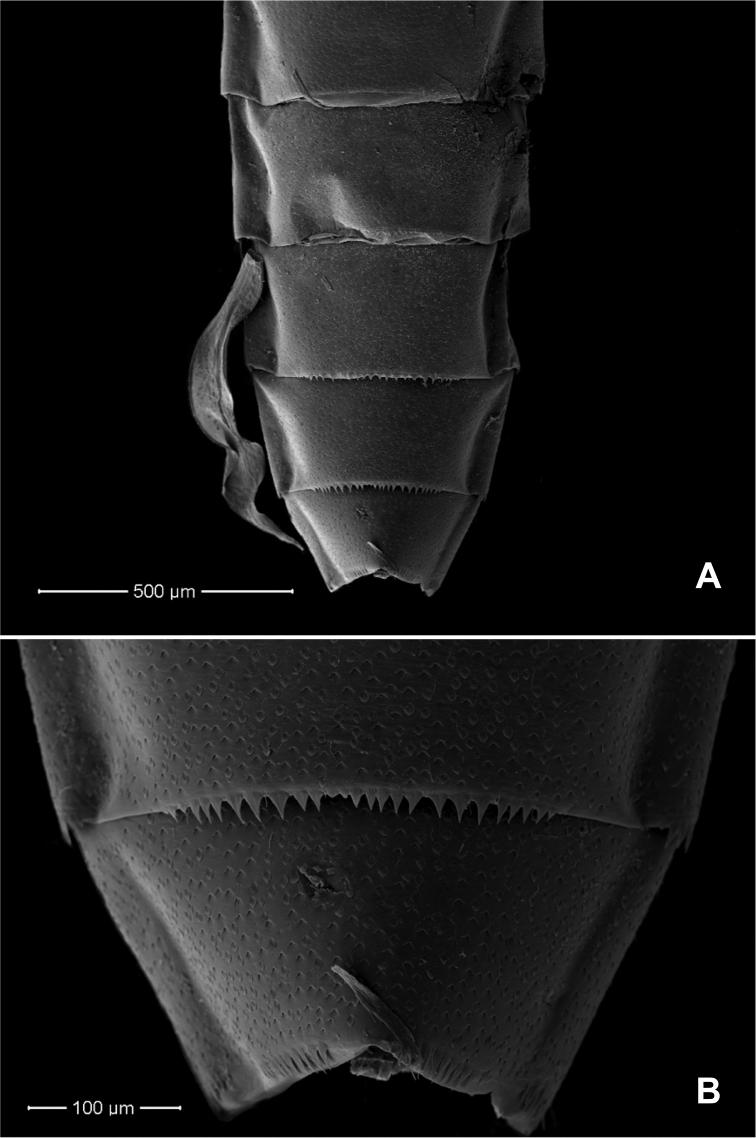
*Procerobaetis totuspinosus* sp. nov., SEMs of tergites **A** tergites V–IX **B** enlargement of tergites VIII–IX.

**Figure 6.**
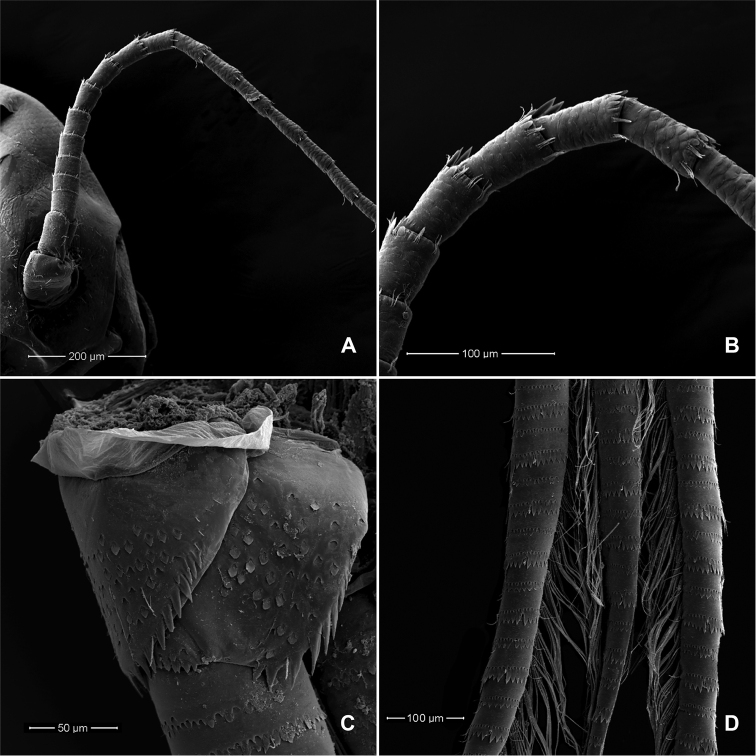
*Procerobaetis totuspinosus* sp. nov., SEMs of larval morphology **A** antenna **B** closer view detail of antenna showing details of lanceolate spines **C** paraproct **D** caudal filaments middle section.

**Figure 7.**
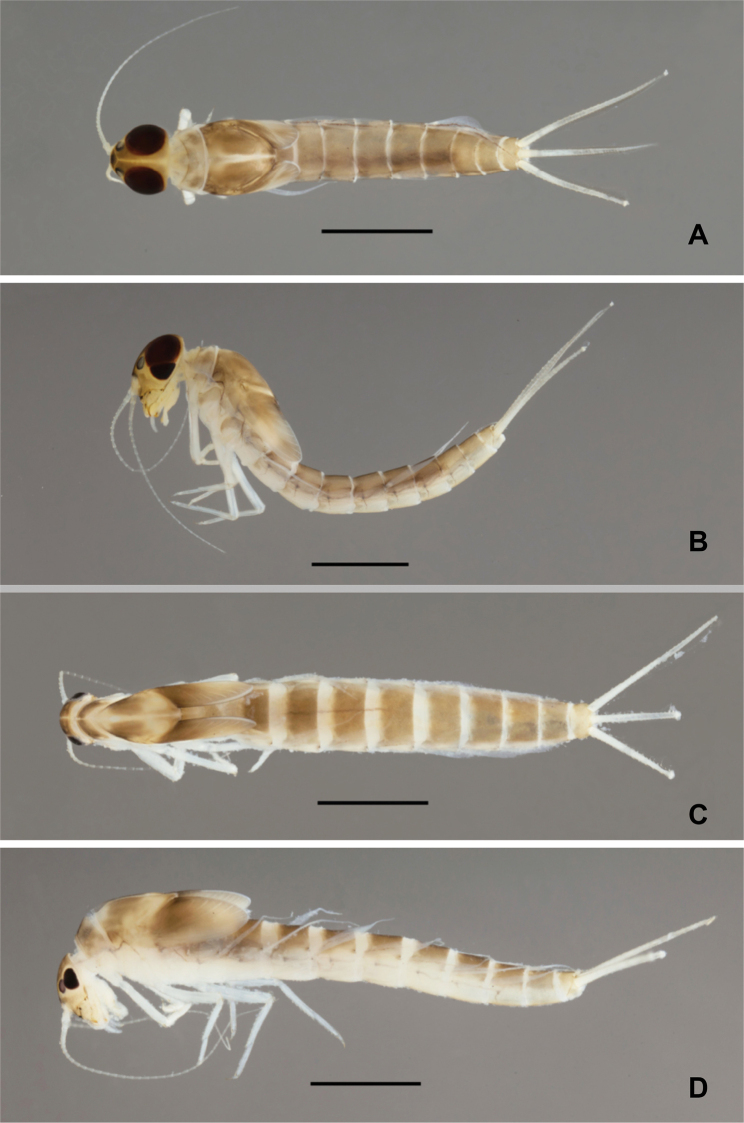
*Procerobaetis totuspinosus* sp. nov., habitus, larvae **A** dorsal view, male **B** lateral view, male **C** dorsal view, female **D** lateral view, female. Scale bars: 1 mm.

Figures 1 , 2 , 3 , 4 , 5 , 6 , 7

##### Materials examined.

***Holotype*.** 1 larva on slide, deposited in ZMKU, Thailand, Chiang Mai, Mae Chaem district, Mae Chaem wadeable river, 18°30'45.3"N, 98°21'23.8"E, 475 m, 16.02.2020, B. Boonsoong leg.

***Paratypes*.** 1 larva on slide, deposited in ZMKU, same data as holotype; 1 larva in alcohol, GBIFCH00673237, deposited in MZL, same data as holotype.

##### Other materials.

6 larvae in alcohol, deposited in ZMKU, same data as holotype. 2 larvae in alcohol, deposited in ZMKU, 1 larva in alcohol, GBIFCH00673238, deposited in MZL, Thailand, Chiang Mai, Mae Rim district, Mae Sa stream, 18°54'39.1"N, 98°55'33.6"E, 355 m, 15.02.2020, B. Boonsoong leg.

##### Description.

**Larva** (Figs 1–7). Body length 4.5–4.6 mm.

***Colouration*** (Fig. 7). Head, thorax and abdomen dorsally brown, head and thorax with bright, median, dorsal suture. Head, thorax and abdomen ventrally light brown. Legs whitish. Caudal filaments whitish.

**Head. *Antenna*** (Figs 2A, 6A, B) approximately 3–4× as long as head length; flagellum with lanceolate spines at apex of each segment, longer at inner lateral margin, increasing in length distally in segment VII–XI and decreasing thereafter.

***Labrum*** (Fig. 1A). Rectangular, length 0.6× maximum width. Distal margin with medial emargination and a small process. Dorsally with many long, stout, simple setae scattered over area, erratically distributed, not arranged in an arc. Ventrally with a marginal row composed of five lateral long, feathered setae and ten anterolateral medial long, bifid, pectinate setae; ventral surface with ca five short, spine-like setae near lateral and anterolateral margins, increasing in length distally.

***Right mandible*** (Fig. 1B, C). Outer and inner sets of denticles with 4 + 1 + 3 denticles. Prostheca stout, arched, shorter than half of canines. Margin between prostheca and mola straight, with a row of medium, stout setae. Setae at apex of mola present.

***Left mandible*** (Fig. 1D, E). Outer and inner sets of denticles with 4 + 3 denticles. Prostheca stout, apically broad, reduced comb-shaped structure. Subtriangular process long and slender, above level of area between prostheca and mola. Denticles of mola apically constricted. Setae at apex of mola absent.

Both mandibles with lateral margins almost straight. Basal half with fine, simple setae scattered over dorsal surface.

***Hypopharynx*** (Fig. 1F). Lingua equal to superlingua, longer than broad, with medial tuft of long, stout setae. Superlingua distally almost straight, lateral margin rounded, with fine, long, simple setae along laterodistal margin.

***Maxilla*** (Fig. 1G). Galea-lacinia with two simple, robust apical setae under crown. Medially with one pectinate, spine-like seta and a row of five long, simple setae increasing in length. Maxillary palp 1.8× as long as length of galea-lacinia with segment I shorter than galea-lacinia; palp segment II 0.5× length of segment I, palp segment III 1.4× length of segment II; setae on maxillary palp fine, simple, scattered over surface of segments I, II and III; apex of last segment rounded.

***Labium*** (Fig. 1H). Glossae basally broad, narrowing toward apex, shorter than paraglossae; inner margin with eight spine-like setae; apex with two long and one medium, robust, pectinate setae; outer margin with six spine-like setae, increasing in length distally; ventral surface with medium, fine, simple, scattered setae. Paraglossae subrectangular, apically curved inward; apex rounded, with two rows of long, robust, simple setae; ventrally two medium, simple setae in anteromedial area; dorsally with a row of four or five long, spine-like setae near inner margin. Labial palp with segment I 0.85× length of segments II and III combined, ventrally scattered with short, fine, simple setae; segment II with very small distomedial expansion, ventrally with scattered short, fine, simple setae, dorsally with a row of four long, spine-like setae; segment III subquadrangular, apex rounded, ventral surface with an arc of five stout, spine-like setae on anteromedially, covered with short spine-like, simple setae and short, fine, simple setae. Mentum distally scattered with fine, simple setae.

**Thorax. *Foreleg*** (Figs 3A, 4). Ratio of foreleg segments 1.4:1.0:0.9:0.3. ***Femur*.** Length 3.7–4.1× maximum width; dorsal margin with a row of six curved, spine-like setae; length of setae 0.23× maximum width of femur; apex rounded, with one pair of spine-like setae (Fig. 4A); many stout, lanceolate, laterally pectinate setae scattered along ventral margin (Fig. 4B); femoral patch absent. ***Tibia*.** Dorsal margin with a row of fine, simple setae; ventral margin with a row of curved, laterally pectinate, spine-like setae, on apex three longer, laterally pectinate, spine-like setae; anterior surface scattered with stout, lanceolate, laterally pectinate setae; patellotibial suture present on basal 1/3 area. ***Tarsus*.** Dorsal margin with a row of fine, simple setae (Fig. 4C); ventral margin with a row of curved, laterally pectinate, spine-like setae, on proximal area with some curved, laterally pectinate, spine-like setae (Fig. 4C), not arranged in a row; tarsal claw (Fig. 4D) elongate, slender, apically pointed, with one row of six or seven larger denticles and many minute denticles, ventral margin at apex straight, with many stripes.

***Middle leg*** (Fig. 3B). As foreleg, but dorsal margin of femur slightly concave.

***Hind leg*** (Fig. 3C). As foreleg, but dorsal margin of femur slightly concave.

**Abdomen. *Tergites*** (Fig. 5). Surface with scattered scales, U-shaped scale bases and micropores. Posterior margin of tergites I–V bare, tergites VI–VIII with triangular spines, tergite IX (Fig. 5B) with triangular spines absent in middle part.

***Gills*** (Fig. 2B–H). Present on segments I–VII; elongate with very long, extended points; margin with very small denticles intercalating fine, simple setae; tracheae limited to main trunk. Gill I (Fig. 2B) as long as length of segments II and III combined, gill II (Fig. 2C) as long as length of segments III and 2/3 of IV combined, gill III (Fig. 2D) as long as length of segments IV and V combined, gill IV (Fig. 2E) as long as length of segments V and VI combined, gill V (Fig. 2F) as long as length of segments VI and VII combined, gill VI (Fig. 2G) as long as length of segments VII and VIII combined, gill VII (Fig. 2H) as long as length of segments VIII–X combined.

***Paraproct*** (Figs 2I, 6C). Posterior margin with nine or ten stout spines; surface scattered with scales and U-shaped scale bases; posterolateral extension (cercotractor) with nine medium, blunt, marginal spines.

***Caudal filaments*** (Fig. 6D). Cerci ca 0.4× body length, median caudal filament ca 0.8× length of cerci.

##### Diagnostic characters.

**Larva.** The main diagnostic characters are: i) the posterior margin of tergites VI–VIII entirely with triangular spines; ii) the maxillary palp is shorter than in other species, with segment I shorter than galea-lacinia; and iii) all gills present extended points as in *P. freitagi*, while in *P. leptobranchius* and *P. petersorum* only gills I and II are apically strongly produced.

##### Winged stages.

Unknown.

##### Etymology.

*Procerobaetis totuspinosus* sp. nov. is a combination of *totus* (derived from the Latin word meaning entire, whole, complete) and *spina* (meaning spine), in reference to the remarkable complete row of triangular spines at the posterior margin of tergites VI–VIII.

##### Distribution.

Chiang Mai province

##### Biological aspects.

**Figure 8.**
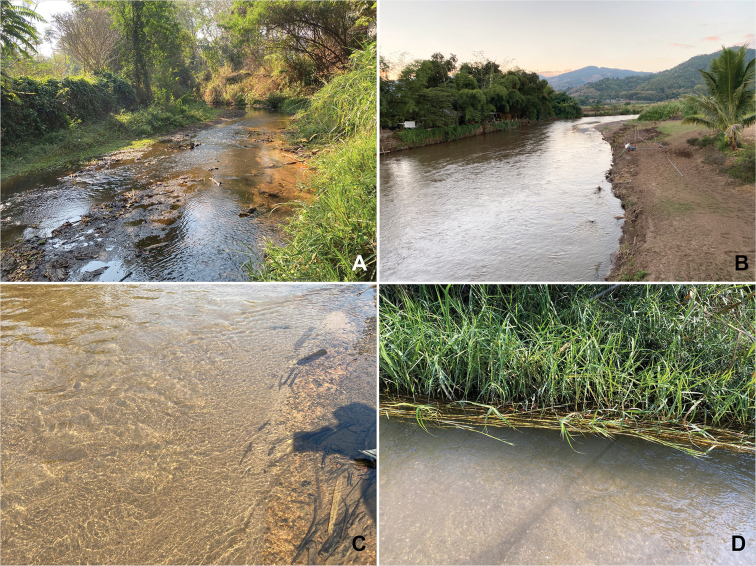
Habitats of *Procerobaetis totuspinosus* sp. nov. larva **A** Mae Sa stream **B** Mae Chaem wadeable river **C** submerged woods with bottom sand and gravel **D** riverbank with vegetation and root.

The specimens were collected in a stream and a wadeable river (Fig. 8) at a range of altitudes (355–475 m a.s.l.). Mae Sa stream is located mostly in residential areas with a partly closed canopy; the substrate was dominated by pebble and gravel, with few patches of leaf litter or dead wood (Fig. 8A). Mae Chaem wadeable river is kind of the submontane type, bordered by farmland and residential areas; its substrate was dominated by sand (Fig. 8B). The larvae of the new species were found in the littoral zone, which was characterised by submerged wood, a sand and gravel bottom (Fig. 8C), and vegetation and roots along the riverbank (Fig. 8D).

##### Molecular analysis.

The Kimura 2-parameter (K2P) analysis revealed interspecific distances (COI) between the new species and the other species of 20–23%. In addition, the interspecific genetic distances of known *Procerobaetis* species varied between13–20% (Table 2).

**Table 2.** Genetic distances (COI) between sequenced specimens, using the Kimura 2-parameter.

		1	2	3	4
1	*Procerobaetis leptobranchius*				
2	*Procerobaetis petersorum*	0.13			
3	*Procerobaetis* sp. C	0.16	0.16		
4	*Procerobaetis freitagi*	0.20	0.19	0.20	
5	*Procerobaetis totuspinosus* sp. nov.	0.22	0.20	0.23	0.20

**Figure 9.**
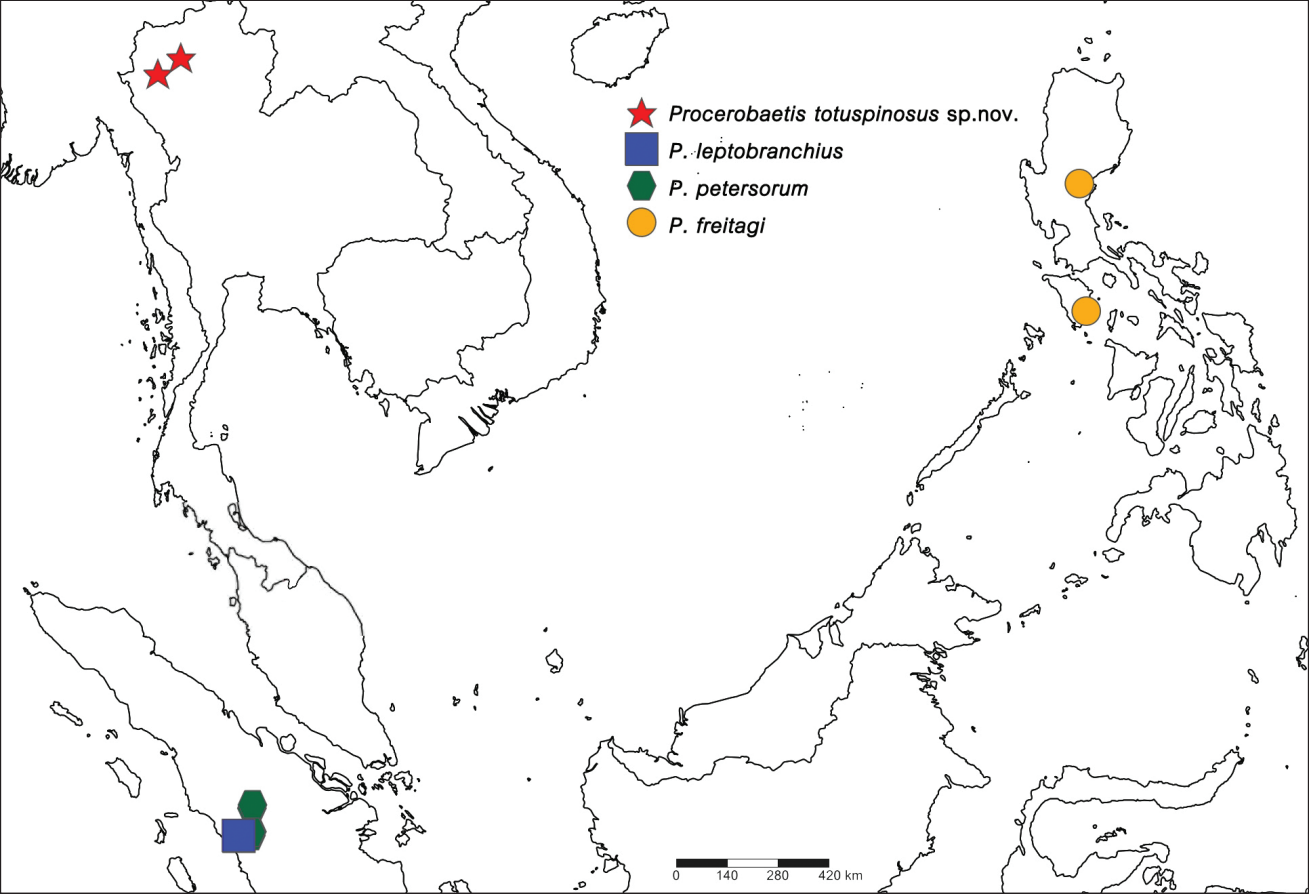
Distribution of genus *Procerobaetis*: *Procerobaetis totuspinosus* sp. nov. (star); *P. leptobranchius* (square); *P. petersorum* (hexagonal); *P. freitagi* (circle).

## Discussion

The genus *Procerobaetis* was established in 2020 by Kaltenbach & Gattolliat. This small genus comprises three species: *Procerobaetis leptobranchius*, *P. petersorum* and *P. freitagi*. It shares with *Nigrobaetis* Kazlauskas in Novikova & Kluge, 1987 and *Alainites* Waltz & McCafferty, 1994 important morphological characters such as: i) body laterally compressed with hypognathous mouthparts, ii) shape of glossae and paraglossae iii) shape of segments II and III of the labial palp (Müller-Liebenau 1984; Kang et al. 1994; Waltz et al. 1994; Gattolliat 2004; Gattolliat et al. 2012; Zrelli et al. 2012). The genus *Procerobaetis* is characterized by very slender, elongate gills with pronounced points and a very long, extended apex at least in gills I and II (Kaltenbach et al. 2020). The relationship of this genus with other closely related genera and non-closely related genera was stated by Kaltenbach et al. 2020.

*Procerobaetis totuspinosus* sp.nov. belongs to the genus *Procerobaetis* based on characters defined by Kaltenbach et al. (2020), and it mainly differs from the three previously known species by the triangular spines on the posterior margin of tergites VI–IX. Both *P. leptobranchius* and *P. petersorum* have triangular spines on the posterior margin of tergite IX only, while *P. freitagi* has triangular spines present on tergites VIII–IX (Kaltenbach et al. 2020). A comparison between this new species and other known *Procerobaetis* species indicates a close morphological similarity between *Procerobaetis totuspinosus* sp. nov. and *P. freitagi* (from the Philippines) in terms of the gill apex shape, the number of medial simple setae of the maxilla, the absence of stout setae on the dorsal margin of the mid tibia and the shape of the ventral margin at the apex of the claw on all legs. However, the new species can be easily distinguished by the presence of triangular spines on tergites VI–IX and a shorter maxillary palp. In addition to the material of the new species described here, we collected a single specimen from a another, distant locality in southern Thailand (Ai Krading stream, Waeng district Narathiwat province). This larva obviously belongs to an undescribed species; however, the lack of sufficient material does not allow a formal description of the new species.

The molecular analysis clearly supports *P. totuspinosus* sp. nov. as a valid species. It is clearly differentiated from other species, as the genetic distances between species range from 20% to 23% (K2P; Table 2); this is much higher than 3.5% which is generally considered as the maximum value for intraspecific divergence (Hebert et al. 2003; Zhou et al. 2010). These results are in line with the genetic distances measured between the three previous *Procerobaetis* species (Kaltenbach et al. 2020). However, this result was based on a single specimen, distances between different populations of the new species obviously remain unknown.

The discovery of *Procerobaetis* in Thailand is the first report of the genus from continental Asia, as all other specimens were collected on islands. The new type locality is situated between Sumatra and the northern Philippines (Fig. 9).

Larvae of *Procerobaetis* should not be overlooked, as they are easily recognisable both in the field and in the lab. However, *Procerobaetis* remains local and rare in Thailand, as in Sumatra and the Philippines, probably due to precise ecological requirements.We may expect a broader distribution including poorly known areas such as continental Malaysia, Laos, Cambodia and Vietnam. Despite intensive field sampling in Brunei and North Kalimantan, allowing recent improvements in the knowledge of the mayfly fauna of Borneo, the genus is still not recorded from this island.

### Key to species of the genus *Procerobaetis*

**Table:** 

1	Triangular spines on posterior margin present only on tergite IX, apex of gills III–VII pointed (Kaltenbach et al. 2020: 10, fig. 3B–H)	**2**
–	Triangular spines on posterior margin present on tergites VI–IX or VIII–IX, apex of gills III–VII with long and extended points (Figs 2B–H, 5)	**3**
2	Dorsal margin of femur (middle and hind legs) slightly concave	***P. leptobranchius***
–	Dorsal margin of femur (middle and hind legs) almost straight	***P. petersorum***
3	Triangular spines on posterior margin present on tergites VIII–IX	***P. freitagi***
–	Triangular spines on posterior margin present on tergites VI–IX (Fig. 5B)	***P. totuspinosus* sp. nov.**

## Supplementary Material

XML Treatment for Procerobaetis totuspinosus
